# Bond-specific fragmentation of oligopeptides via electronic stopping of swift heavy ions in molecular films

**DOI:** 10.1038/s41598-022-21744-w

**Published:** 2022-10-26

**Authors:** P. Schneider, P. Keller, I. Schubert, M. Bender, C. Trautmann, M. Dürr

**Affiliations:** 1 Institut für Angewandte Physik and Zentrum für Materialforschung, Justus-Liebig-Universität Giessen, Heinrich-Buff-Ring 16, 35392 Giessen, Germany; 2 GSI Helmholtzzentrum für Schwerionenforschung GmbH, Planckstrasse 1, 64291 Darmstadt, Germany; 3 Fachbereich Ingenieurwissenschaften, Hochschule RheinMain, Kurt-Schumacher-Ring 18, 65197 Wiesbaden, Germany; 4 Fachbereich Materialwissenschaften, Technische Universität Darmstadt, Alarich-Weiss-Strasse 2, 64287 Darmstadt, Germany

**Keywords:** Condensed-matter physics, Biomaterials

## Abstract

Highly bond-specific fragmentation of oligopeptides induced by swift heavy ion (SHI) irradiation was investigated by means of mass spectrometry. In pronounced contrast to measurements of samples irradiated with keV ions, oligopeptides which were exposed to 946 MeV Au ions show a high abundance of specific fragments. The highly bond-specific nature of SHI-induced fragmentation is attributed to electronic stopping as the most relevant energy loss mechanism for SHI in the oligopeptide samples in combination with the subsequent coupling between the excited electronic and the atomic subsystem. Fragmentation induced by SHI is observed to be further influenced by the structure of the oligopeptides, suggesting that electronic excitation and/or the electronic-vibrational coupling depend on the details of the molecular structure.

## Introduction

**Figure 1:**
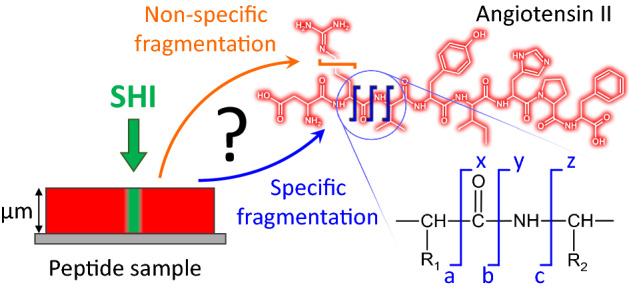
Irradiation of oligopeptide films with swift heavy ions leads to fragmentation of the sample molecules in the vicinity of the ion trajectory (schematic on the left, image not to scale, ion track diameter in the range of some ten nanometers, see below). This SHI-induced fragmentation may either involve the bonds of the peptide backbone (specific fragmentation, examples indicated by blue lines) or arbitrary bonds of the amino acid side chains (non-specific fragmentation, example indicated by the orange line).

Single swift heavy ions (SHI) are known to uniquely alter the properties of solid materials by means of bond breaking and defect creation. This leads to nanostructures both at the surface and in the bulk of inorganic and organic materials^1–3^ and can be utilized, e.g., for pore formation by etching the altered material along the SHI tracks^4–6^. The formation of ion tracks in inorganic material is well described by the thermal spike model^7–9^: the impinging SHI first leads to excitation of the electronic system of the sample ($\le 1$ fs); on a much longer timescale ($\approx$ ps), energy is transferred to the atomic subsystem via electron-phonon coupling resulting in a thermal spike and local melting^2,10^. In organic materials, the melting criteria is not necessarily relevant as the impinging ion and the induced electron cascade can lead to cleavage of single covalent bonds and radiolysis of the molecules^11,12^. In particular, in the case of complex molecules with a variety of different chemical bonds, the question arises whether fragmentation induced by SHI can be bond-specific and which parameters influence the cleavage probability for a given bond. Despite its relevance both for a fundamental understanding of SHI interaction with organic matter as well as for applications such as cancer therapy and biomaterials development^13–15^, this question has not yet been addressed in full detail. Oligopeptides with their peptide backbone and a multitude of functional groups in the side chains are predestinated to study such bond-specific fragmentation in organic molecules (Fig. 1).

In this contribution, we employ Desorption/Ionization Induced by Neutral SO$_2$ Clusters (DINeC) in combination with mass spectrometry (MS) for the investigation of SHI-induced fragment formation in oligopeptide films. DINeC is an extremely soft desorption technique which can be applied to various substance classes including peptides, lipids, dyes, and ionic liquids^16–19^. In combination with mass spectrometry, it represents an analytical tool with high chemical sensitivity which allows for direct, unambiguous characterization of different types of fragmentation induced in organic molecules^20,21^. We find that SHI irradiation of oligopeptides predominantly leads to specific fragmentation of the peptide backbone of the intact molecule (Fig. 1), in clear contrast to similar experiments with projectile ions in the keV energy range^21^. This indicates that the fragmentation is largely influenced by the underlying mechanism of energy deposition, i.e., electronic stopping in the case of SHI versus nuclear stopping in the case of keV ions. Moreover, sensitivity of a given molecule on SHI irradiation as well as the number of different fragment species observed depend on the oligopeptide investigated, suggesting an influence of the molecular structure on the details of ion-molecule interaction and SHI-induced fragmentation.

## Experimental

**Figure 2:**
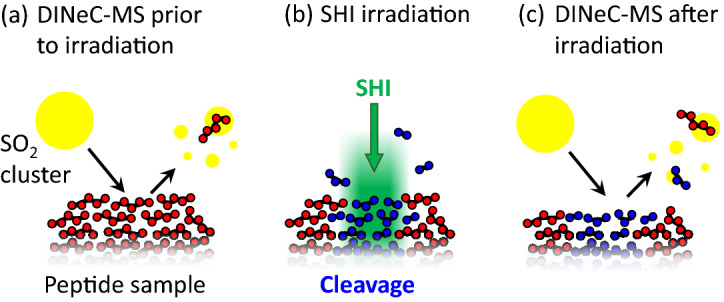
Schematic representation of the experimental procedure. (**a**) Freshly prepared oligopeptide films were first characterized by means of DINeC-MS. (**b**) Samples were then irradiated with 946 MeV Au ions at GSI, leading to cleavage of covalent bonds in the molecules. (**c**) Subsequent DINeC analysis of the irradiated samples reveals SHI-induced fragments which remain in the sample and have not been sputtered during SHI-irradiation. Schematics are not to scale.

The experimental procedure applied for all samples in this study is schematically depicted in Fig. 2: Directly after preparation, the fresh samples were characterized by means of DINeC-MS. Subsequently, the samples were transferred under ambient conditions to the GSI Helmholtzzentrum für Schwerionenforschung, Darmstadt, Germany, where SHI irradiation was performed. After irradiation, the samples were transferred back (ambient conditions) to the DINeC-MS apparatus in order to analyze them with respect to ion-induced fragmentation. In this way, the fragments which are induced and remain in the topmost layers of the sample are analyzed, different to online secondary ion or secondary neutral mass spectrometry experiments (SIMS/SNMS), which analyze the fragments which are sputtered during ion impact^22^.

Oligopeptide samples were prepared by drop casting 30 $\mu$L of aqueous solution of the respective molecule (${c=10^{-3}}$ mol/L) on a silicon wafer (about $1 \times 1$ cm$^2$ in size) covered with the natural oxide layer^23^. The wafers were then dried in a desiccator leading to molecular films of different thickness up to some micrometers^24^. The resulting sample spots were between 3 and 8 mm in diameter, depending on the oligopeptide used. Three different oligopeptides were investigated: angiotensin II, bradykinin, and neurotensin. All three of them show specific biological activity; they clearly differ in their amino acid sequences and have been widely used in mass spectrometric investigations. For the bradykinin films, the respective acetate salt was used as starting material.

The DINeC-MS apparatus is based on a commercially available ion trap mass spectrometer (amaZon speed, Bruker Daltonik GmbH, Bremen, Germany) which is equipped with a customer-built DINeC ion source. SO$_2$ clusters are created by expanding a pressurized gas mixture ($\approx 3 \%$ SO$_2$ in He, $p\approx 15$ bar) into the vacuum chamber ($p = 10^{-6}$ mbar) through a pulsed nozzle (effective opening time about 500 $\mu$s, pulse frequency $\approx 2$ Hz). The resulting SO$_{2}$ clusters are of about $10^3$ to $10^4$ molecules in size and have a narrow velocity distribution around 1600 m/s^25^. This converts into a constant energy density of the clusters upon collision with the sample which is below 1 eV per molecule; the latter is typically seen as the threshold for sputtering of organic material in secondary ion mass spectrometry using cluster projectiles^26^. Further details on the experimental setup have been published previously^17^.

Sample irradiation was performed at the M3 beamline of the universal linear accelerator (UNILAC) at GSI with Au$^{26+}$ ions (4.8 MeV/u which corresponds to 946 MeV kinetic energy per ion). The flux was between $3\times 10^{7}$ to $3\times 10^{8}$ ions/(s cm$^2$). Applied fluences ranged from $5\times 10^{10}$ to $6\times 10^{11}$ ions/cm$^2$. Samples were stored and transferred under ambient conditions in a dark container to prevent photo-induced sample degradation.

## Results

**Figure 3:**
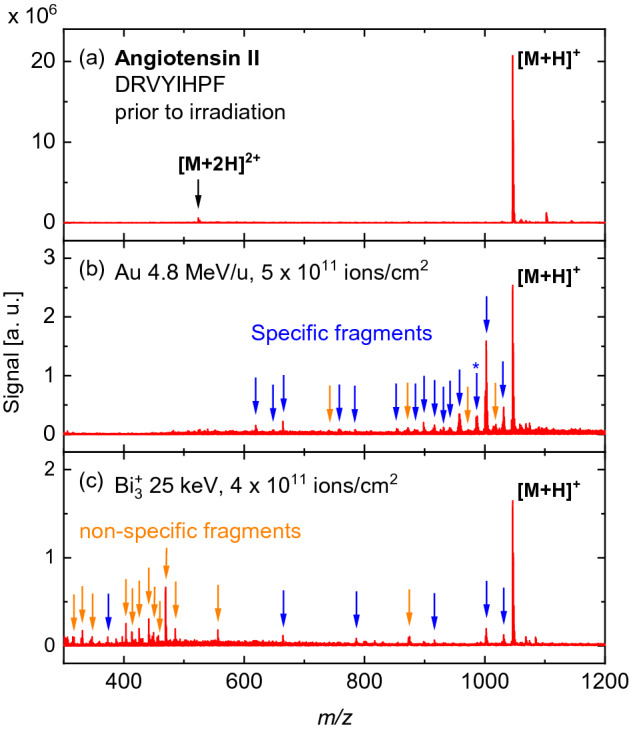
DINeC mass spectra (positive ion mode) from (**a**) a freshly prepared angiotensin II sample, (**b**) the same sample irradiated with 946 MeV Au ions, and (**c**) an angiotensin II sample irradiated with 25 keV Bi$_3^+$ ions. Fragment peaks with signal intensities higher than $\approx 3$% of the signal of the intact molecule are labeled with arrows. In the spectrum of the fresh sample, no fragment peaks are present. In the case of SHI irradiation, most peaks can be assigned to specific fragments (blue arrows). A fragment corresponding to double cleavage of the peptide backbone is also observed (indicated by a star). In contrast, the spectrum from the sample irradiated with keV ions shows mostly non-specific fragment peaks (orange arrows). In the *m*/*z* range below 300, no significant fragment peaks were observed (compare Fig. S2 in the Supporting Information). The difference in the noise level between (**b**) and (**c**) is within the fluctuation from sample to sample. The spectrum in (**c**) has been adopted from Ref.^21^.

A DINeC mass spectrum of a fresh angiotensin II film is depicted in Fig. 3a. A single major peak is detected at $m/z=1046.5$, corresponding to intact, protonated angiotensin II, [M+H]$^+$. A peak associated with the doubly charged molecule, [M+2H]$^{2+}$, is observed at $m/z=523.8$. A few smaller peaks at *m*/*z* values slightly higher than 1047 are attributed to angiotensin II molecules with alkali ions as adducts, i.e., [M+Na]$^+$, [M+K]$^+$, etc.^27^. Most importantly, no significant peaks are observed at *m*/*z* values below 1047. First, this implies that no fragments are present in the fresh angiotensin II film. Second, no fragmentation occurs during the SO$_2$-cluster-induced desorption process.

Figure 3b presents a mass spectrum of the same sample after irradiation with $5\times 10^{11}$ Au ions/cm$^2$, showing a significant decrease of the intensity of the peak corresponding to the intact molecule. Moreover, various additional peaks are observed, which were not detected prior to irradiation. Most of these peaks can be assigned to specific fragments resulting from breaking one of the bonds in the backbone of the original molecule (Fig. 1, compare also Tab. S1 in the Supporting Information). The peak at $m/z = 987.5$ (labeled with a star) is even assigned to a specific fragment resulting from double cleavage of the peptide backbone. Specific fragments are well known from various other fragmentation methods. In particular, they play a major role in the context of peptide/protein identification by means of tandem mass spectrometry^28^; in this work, we have adopted the fragment nomenclature used in that type of studies^29,30^. The spectra of SHI-irradiated angiotensin II show practically no fragments other than specific ones; the few peaks corresponding to non-specific fragments exhibit very low intensities.

In order to rule out the possibility that the observed fragmentation is due to macroscopic beam-induced temperature effects, we performed the irradiation experiments with different SHI fluxes. The resulting mass spectra are generally very similar, both qualitatively and quantitatively (compare also Fig. S3 in the Supporting Information). We thus exclude effects due to beam-induced macroscopic heating of the samples and attribute the specific fragmentation to the direct interaction between SHI and the angiotensin II molecules.

The results for SHI-induced fragmentation of angiotensin II are compared to a mass spectrum from an angiotensin II sample which was irradiated with 25 keV Bi$_3^+$ ions, shown in Fig. 3c^21^. Most striking is the difference in the fragmentation pattern despite the similar reduction of the peak associated with the intact molecule: The sample exposed to keV ions predominantly shows non-specific fragmentation and only a few specific fragments. In contrast, specific fragmentation is clearly dominant in the case of SHI. On a first glance, this observation seems contradictory when considering the comparably high kinetic energy and the concomitant high stopping power of about 10 keV/nm of the swift Au ions in organic material (Fig. S4) which has to be compared to a stopping power of about 2 keV/nm for the Bi$^+_3$ ions in comparable material^31,32^. However, one has to take into account the different excitation mechanisms which are operative for SHI and keV ions. In the discussion below, we will elaborate on how the rather controlled peptide fragmentation induced by SHI irradiation is closely related to electronic stopping as the dominant excitation mechanism. In contrast, nuclear stopping is predominant in the case of the keV ions leading to non-specific fragmentation.

**Figure 4:**
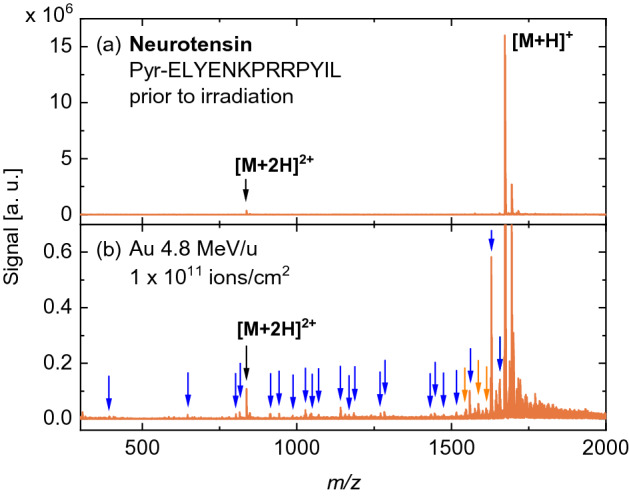
DINeC mass spectra from (**a**) a freshly prepared neurotensin sample and (**b**) the same sample irradiated with 946 MeV Au ions. In the mass spectrum of the irradiated sample, a multitude of fragment peaks is observed. Almost all of these peaks can be assigned to specific fragments of the molecule (Tab. S2). The number of different fragment peaks is significantly higher when compared to spectra of irradiated angiotensin II samples. The peak at $m/z = 1694.7$, which is present in both spectra, is assigned to [M+Na]$^+$. The signal intensity close to the peak of the intact molecule ($m/z > 1673$) is comparable to the measurement before irradiation but appears more pronounced in (**b**) due to the smaller signal scale.

In order to investigate how the molecular structure influences the fragmentation characteristics, the experiments were performed with two additional oligopeptides. In Fig. 4, DINeC mass spectra from a neurotensin sample prior to and after irradiation are shown. As with angiotensin II, no fragments are observed prior to irradiation. After SHI irradiation, the peak corresponding to the intact molecule is decreased and additional peaks appear, almost all corresponding to specific backbone fragments. In general, the number of fragments observed for irradiated neurotensin is higher than for angiotensin II.

**Figure 5:**
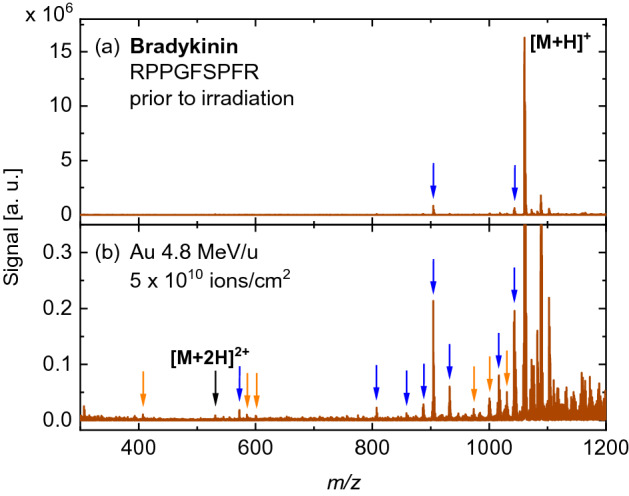
DINeC mass spectra from (**a**) a freshly prepared bradykinin sample and (**b**) the same sample irradiated with Au ions (946 MeV). Fragments observed prior to irradiation at $m/z = 904.3$ and 1043.5 are due to the loss of one of the molecules’ terminal arginine groups or an OH group, respectively, when the film is exposed to ambient atmosphere. SHI irradiation leads to additional fragmentation (Tab. S3); overall, fragment peaks are less abundant when compared to DINeC mass spectra of angiotensin II or neurotensin. The signal intensity close to the peak of the intact molecule ($m/z > 1060$) is comparable to the measurement before irradiation but appears more pronounced in (**b**) due to the smaller signal scale.

Similarly, mass spectra of a bradykinin sample prior to and after irradiation are depicted in Fig. 5. In that case, the spectrum prior to irradiation shows already two peaks corresponding to specific fragments which are formed when bradykinin films are prepared and stored under ambient conditions. SHI irradiation then leads to additional fragmentation. When compared to angiotensin II, the number of fragment peaks observed in the spectrum from the irradiated bradykinin sample is significantly lower; still, the majority of peaks corresponds to specific fragments (compare Tab. S3).

**Figure 6:**
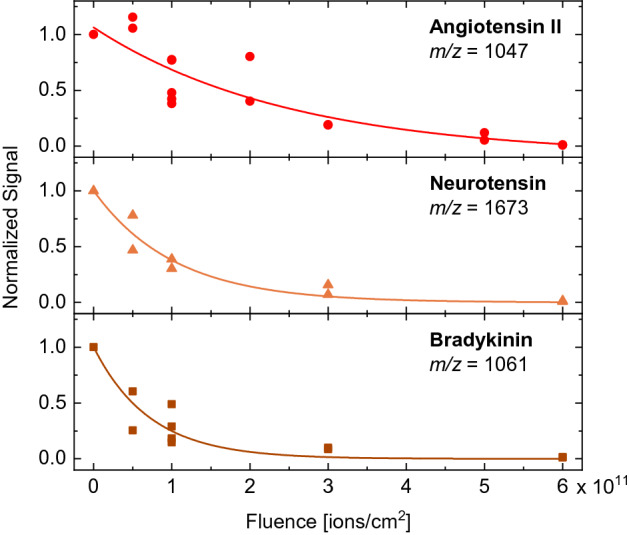
Signal intensity of the peaks associated with the intact oligopeptides as a function of SHI fluence for (**a**) angiotensin II, (**b**) neurotensin, and (**c**) bradykinin. Each data point represents the measurement of a separate sample which was irradiated with the given fluence; the signal of each irradiated sample was referenced to the signal of the same sample prior to irradiation. Exponential fits to the experimental data are represented by solid lines. The variance in the experimental data may be in part explained by variations of the sample position probed during the DINeC measurements prior to and after irradiation as well as by some inhomogeneity of the irradiation.

For all three molecules, the signal intensity *I* of the peak corresponding to the intact analyte molecule is shown in Fig. 6 as a function of the Au-ion fluence *F*. In all cases, the signal decreases with increasing ion fluence due to accumulation of damage in the sample. The evolution is well described by an exponential decay function. When comparing the results for different oligopeptides, we observe that the decrease in signal intensity as a function of SHI fluence is strongest for bradykinin while angiotensin II shows the weakest decay. This trend is also reflected in the cross sections $\sigma$ which were deduced from the exponential fits $I(F)=I_0 \exp (-\sigma F)$: $\sigma _\mathrm{angiotensin II} = 400$ nm$^2$, $\sigma _\mathrm{neurotensin} = 970$ nm$^2$, and $\sigma _\mathrm{bradykinin} = 1390$ nm$^2$. At the same time, bradykinin also shows the lowest abundance of fragment peaks which seems contradictory at first glance. However, one has to keep in mind that mass spectrometry can only record ions and not neutral molecules. In particular, bradykinin is protonated with a high probability via the terminal arginine units, which exhibit a pronounced basic character, i.e., the ability to take up a proton. These terminal arginine units are especially prone to fragmentation as indicated by the intensive peaks at $m/z=904$ and 932 in Fig. 5. Therefore, some fragments, although present in the sample, might not be observed in the mass spectra due to loss of the arginine units and concomitant low ionization probability.

## Discussion

In the following, we want to discuss the dependence of signal intensity on SHI fluence as well as the fragmentation patterns for the different oligopeptides in more detail. In particular, the high abundance of specific fragments observed in the mass spectra of SHI irradiated samples seems to be surprising: Generally, specific fragmentation is prominently observed in low energetic dissociation processes, such as low-energy collision induced dissociation with a typical collision energy below 100 eV^33,34^. With increasing excitation energy in the keV range, non-specific fragmentation becomes dominant, e.g., in secondary ion mass spectrometry or high-energy collision induced dissociation^33,35^. Therefore, when considering only the high kinetic energy and energy loss of SHI projectiles interacting with films of fragile oligopeptides, non-specific fragmentation is expected to be predominant. Given the high energy involved in the process, also the pronounced influence of the molecular structure comes to some surprise at first glance.

**Figure 7:**
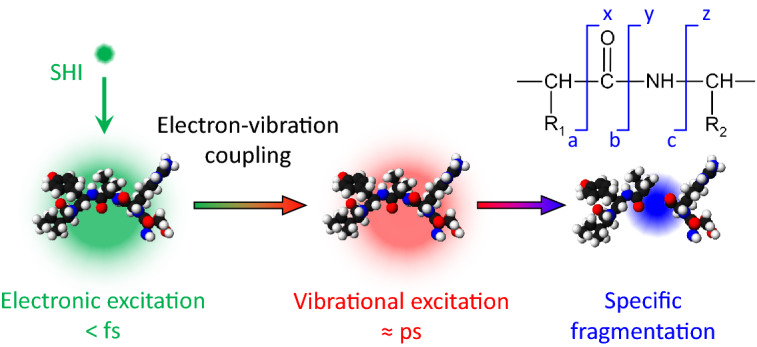
Schematic illustration of SHI-induced excitation and fragmentation of oligopeptides via coupling between the electronic and vibrational excitation. The impinging SHI leads to excitation of the electronic subsystem only (sub-femtosecond timescale). The electronic excitation is converted into highly vibrationally excited molecules on the picosecond timescale. As a consequence, the covalent bonds most prone to the underlying fragmentation mechanism, i.e., in this case, the peptide bonds of the molecules’ backbone, are cleaved leading to the specific fragments observed in the mass spectra.

This simple picture, however, implies that energy transfer from the projectile to the atomic degrees of freedom of the molecule occurs directly and locally as it is the case for keV ions in the nuclear stopping regime which transfer the energy directly into the atomic subsystem of the molecule via elastic collisions. However, the dominant energy loss mechanism for swift heavy ions in condensed matter is electronic stopping, i.e., in a first step the electronic subsystem of the molecules is excited^36^. In the organic molecules investigated, this electronic excitation may lead in two ways to the observed fragmentation: (i) Electronic excitation of the molecules by the SHI or the resulting electron cascade can lead to a direct cleavage of the covalent bonds when antibonding states are occupied. This requires that the lifetime of the excited state is long enough for the involved atoms to gain the momentum necessary for dissociation of the chemical bond. Such a direct dissociation is often operative for UV-light induced photodissociation of smaller molecules in the gas phase^37,38^. (ii) The vibrational degrees of freedom of the molecules can be excited subsequent to electronic excitation, e.g., if the atomic subsystem does not reach enough momentum for direct fragmentation during the electronic excitation, the kinetic energy gained on the excited potential energy surface will be transformed into vibrational excitation. Friction-like coupling between the SHI-excited electrons and the vibrational degrees of freedom can further lead to vibrational excitation of the molecules. Depending on the degree of intramolecular vibrational energy distribution, these vibrational excitations can be distributed over the whole molecule. As a consequence, the bonds which are most prone to fragmentation under these conditions will be cleaved (compare Fig. 7). In the oligopeptides investigated, these are then the peptide bonds of the molecules’ backbone. Potentially, this peptide cleavage proceeds via secondary processes such as the interaction with a mobile proton^39^.

For fragmentation *directly* induced by electronic excitation, one expects not only the peptide bonds to be cleaved but also bonds of the side chains. As an example, the phenol group in tyrosine is prone to dissociation after electronic excitation by UV light^38^, but also other functional groups of the side chains can be cleaved^37^. In the analysis of the SHI-induced fragments of the three oligopeptides of this investigation, we do not find such fragments. We therefore conclude that electronic excitation followed by vibrational excitation is the main process leading to SHI-induced fragmentation of oligopeptides. Similar to this process, collision induced dissociation in the low energy regime ($<100$ eV) leads predominantly to specific fragments^33,34^; also for UV photodissociation of oligopeptides, such an indirect process is discussed^40,41^.

In this picture, the distribution of the vibrational excitation in one single molecule will depend on the details of the molecular structure. It is thus in line with the observation that both the fragmentation patterns in the mass spectra as well as the abundance of intact molecules as a function of SHI fluence depend on the respective molecule, i.e., on the underlying amino acid sequence.

Local excitation of vibrational modes as a result of SHI-molecule-interaction as described above can be seen as an intermediate towards a local temperature increase around the ion trajectory. For the systems investigated, apparently the energy spread is too large in order to reach a temperature high enough to induce both specific and non-specific fragmentation distributed over the whole molecule in a quantity which can be measured in our experiment. The relatively large cross sections obtained for all three molecules in comparison to typical ion track radii observed in organic material^42–44^ indicate that the electron cascade and its spatial distribution play an important role for the excitation/fragmentation process.

## Conclusion

In conclusion, we have observed ion-induced excitation and fragmentation of oligopeptides to be strongly influenced by the nature of the stopping mechanism which is operative: Ions in the keV regime interact directly with the atomic subsystem which implies a strong local correlation between the interaction site and the broken bond. As a consequence, a high abundance of non-specific fragments is observed. In contrast, SHI first excite the electronic subsystem of the sample. In organic samples, this electronic excitation and the concomitant electron cascade may either lead to direct bond cleavage or fragmentation induced by the excitation of the vibrational degrees of freedom following the initial electronic excitation. From our results, we conclude the latter process to be the main channel for the specific fragmentation. It explains the counterintuitive observation that projectile ions in the MeV to GeV energy regime lead to a much more bond-specific fragmentation pattern when compared to ions in the keV range.

## Supplementary Information

Supplementary Information.

## Data Availability

The datasets and materials generated during the current study are available from the corresponding author on reasonable request.
