# Supplementary material for: Bond-specific fragmentation of oligopeptides via electronic stopping of swift heavy ions in molecular films
Source: Sci Rep. 2022 Oct 26;12:17975. doi: 10.1038/s41598-022-21744-w (PMC9605986; doi:10.1038/s41598-022-21744-w)
Supplement: Supplementary file 1 — Supplementary Information. [file 41598_2022_21744_MOESM1_ESM.pdf]

Supporting Information for:

Bond-specific fragmentation of oligopeptides via electronic stopping of swift heavy ions in molecular films

P. Schneider[1], P. Keller[1], I. Schubert[2], M. Bender[2,3], C. Trautmann[2,4] and M. Dürr[1,*]

[1]*Institut für Angewandte Physik and Zentrum für Materialforschung, Justus-Liebig-Universität Giessen, Heinrich-Buff-Ring 16, D-35392 Giessen, Germany*

[2]*GSI Helmholtzzentrum für Schwerionenforschung GmbH, Planckstrasse 1, D-64291 Darmstadt, Germany*

[3]*Fachbereich Ingenieurwissenschaften, Hochschule RheinMain, Kurt-Schumacher-Ring 18, D-65197 Wiesbaden*

[4]*Fachbereich Materialwissenschaften, Technische Universität Darmstadt, Alarich-Weiss-Strasse 2, D-64287 Darmstadt, Germany*

[*]*Corresponding author: michael.duerr@ap.physik.uni-giessen.de*

Table S1: Fragment species observed in SHI-irradiated angiotensin II samples. Specific fragments are labeled according to the nomenclature introduced by Biemann [1, 2]. Fragments resulting from double cleavage of the peptide backbone are labeled according to Fig. S1. Reactive fragments are frequently observed at $\Delta m/z = +2$ with respect to their nominal mass; they are denoted by +2H indicating the uptake of two further hydrogen atoms. The table includes both specific and non-specific fragments which are not highlighted in Fig. 3 in the main article since they exhibit intensities below 3% of the intensity of the peak corresponding to the intact molecule.

$\boldsymbol{m/z}$	**Fragment species**
506.3	a_4
523.8	$[M+2H]^{2+}$
526.3	*non-specific*
534.3	b_4
539.4	x_4
551.4	c_4
613.4	*non-specific*
619.3	a_5
647.3 / 649.3	b_5 / b_5+2H
664.4	c_5
741.4	*non-specific*
756.4 / 758.4	a_6 / a_6+2H
758.4	z_6
784.4	b_6
853.4 / 855.4	a_7 / a_7+2H
872.5	*non-specific*
883.5	b_7+2H
898.4	c_7
916.5	z_7+2H
926.5	*non-specific*

931.5	y_7
942.5	x_7-NH
957.5	x_7
972.5	*non-specific*
985.5 / 987.5	a_8-z_8+H / a_8-z_8+3H
1000.5 / 1002.5	a_8 / a_8+2H
1018.5	*non-specific*
1031.5	z_8+2H
1046.5	$[M+H]^+$

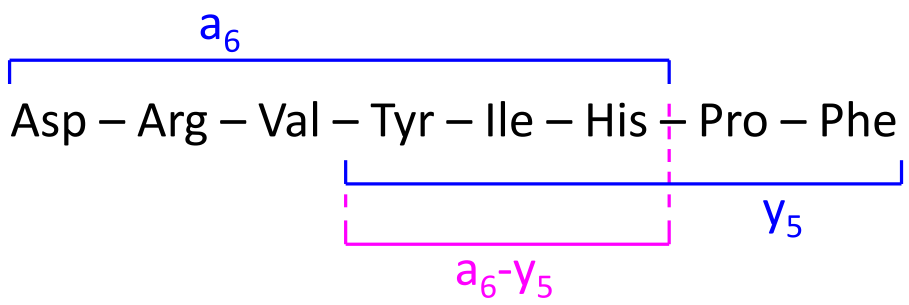

Figure S1: Example for the nomenclature of fragments resulting from double cleavage of the peptide backbone. The fragment name is composed of the names of the fragments corresponding to the two cleavage sites.

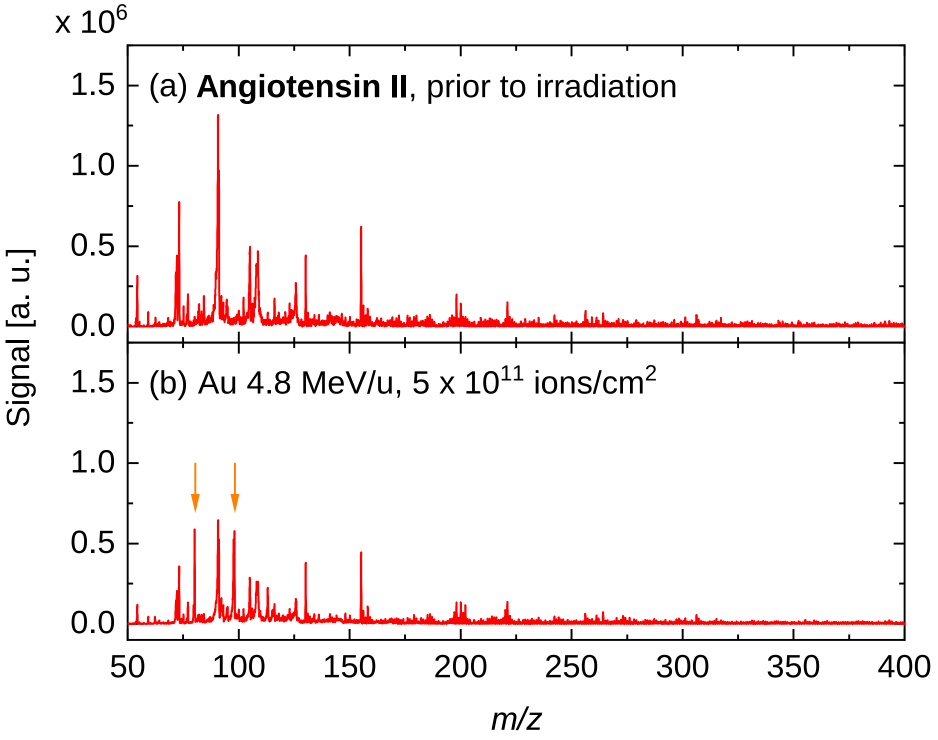

Figure S2: DINeC mass spectra of angiotensin II in the low m/z range between 50 and 300 (a) prior to, and (b) after SHI irradiation (compare Figs. 3 (a) and (b) in the main article). Most peaks observed in the irradiated sample are already present in the measurement prior to irradiation. Two major additional peaks are observed after SHI irradiation (orange arrows) which are also observed in bradykinin samples irradiated together with this sample. Moreover, samples from other irradiation series do not show these peaks at all. They probably have to be attributed to sample contamination during transfer or irradiation. In particular, no significant fragment peaks are observed in this m/z range.

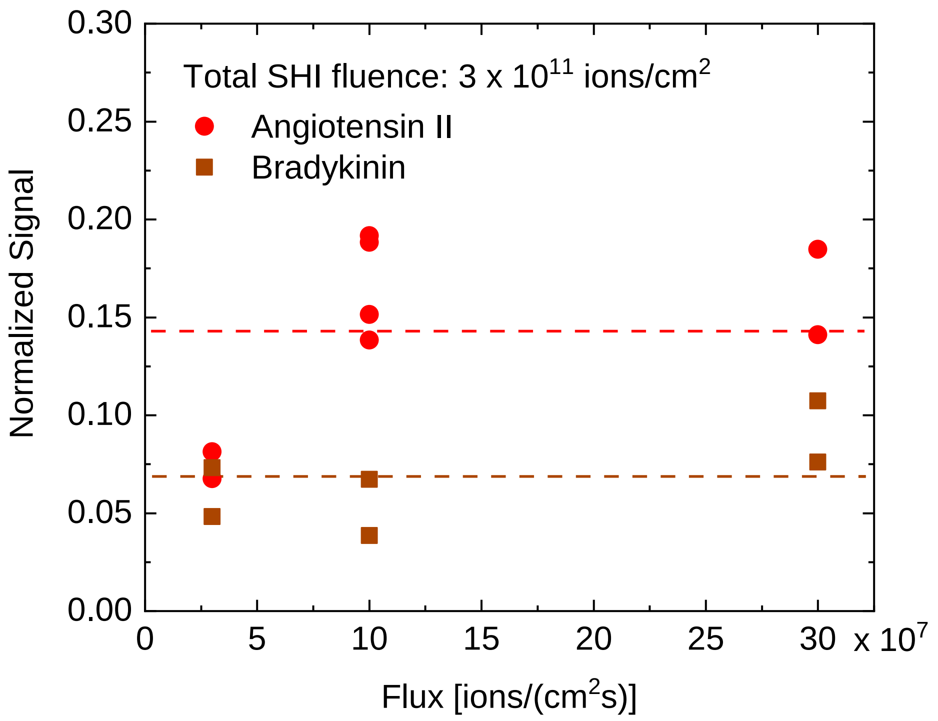

Figure S3: Signal intensity (peak maximum) of the peaks associated with intact angiotensin II and bradykinin molecules in SHI-irradiated samples as a function of applied SHI flux. Total accumulated fluence was 3×10^{11} ions/cm^2 for all samples; the signal of each irradiated sample was normalized to the signal of the same sample prior to irradiation. The signal is largely independent of the flux. Dashed lines correspond to the respective mean values of the relative signal. For angiotensin II, a rather low signal is observed in the case of 3×10^7 ions/cm^2 which might be in part explained by some inhomogeneity of the irradiation resulting in a in total higher effective fluence on the sample. Most importantly, higher SHI flux does not to lead to higher damage (i.e., lower signal intensity of the intact molecules) in the sample. Therefore, the fragmentation observed in the irradiated samples is attributed to direct SHI-molecule-interaction rather than secondary effects such as heating of the sample.

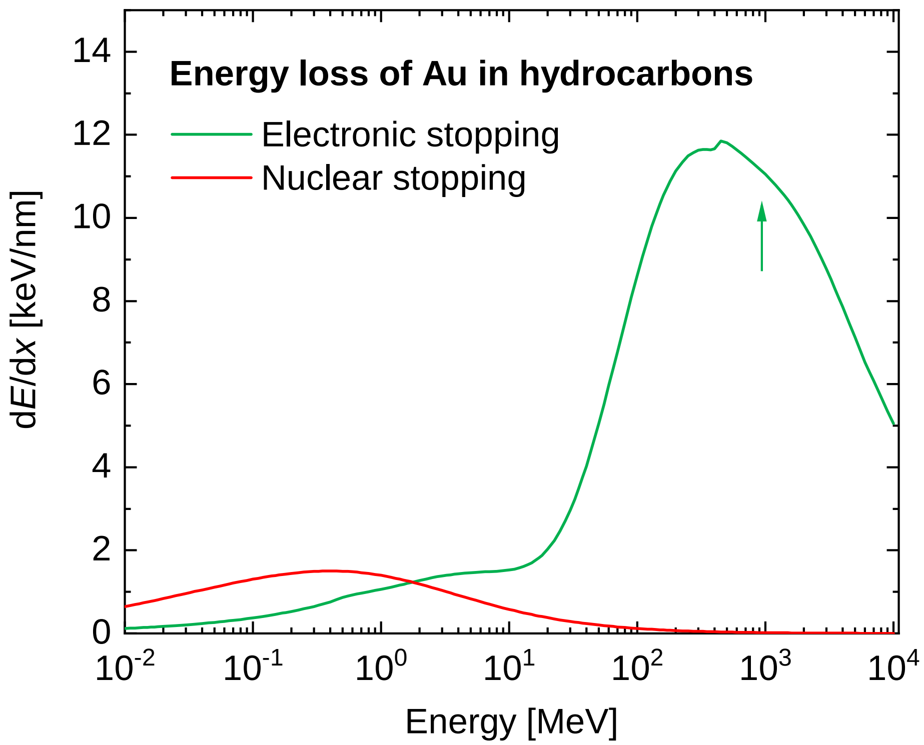

Figure S4: Stopping power of Au ions in hydrocarbon material (0.8 g/cm^3) as a function of kinetic energy according to SRIM-2013 calculations [3]. The Au ions used for irradiation in this study exhibit a kinetic energy of 946 MeV (indicated by the arrow). The initial energy loss amounts to dE/d$x \approx 11$ keV/nm and is attributed almost exclusively to electronic stopping. The range of the ions in hydrocarbon material is about 100 μm, i.e., much higher than the thickness of the oligopeptide films investigated.

Table S2: Fragment species observed in SHI-irradiated neurotensin samples. The nomenclature is the same as used in Tab. S1.

m/z	**Fragment species**
393.2	z_3+2H
407.2	*non-specific*
571.3	*non-specific*
585.3	*non-specific*
602.3	*non-specific*
646.4	z_5+2H
661.4	y_5
689.4	x_5+2H
757.3	*non-specific*
779.9	c_{12}+H^+
802.5	z_6+2H
814.9	a_{13}+2H+H^+
828.3	a_7
836.9	$[M+2H]^{2+}$
845.4	x_6+2H
847.9	$[M+H+Na]^{2+}$
873.4	c_7
914.5	y_7
942.5	x_7+2H
986.5	a_8+2H
999.5	*non-specific*
1012.5	b_8
1023.6	*non-specific*
1027.6	z_8+2H
1042.6	y_8

1046.5	angiotensin II
1068.6 / 1070.6	x_8 / x_8+2H
1139.6 / 1141.6	z_9 / z_9+2H
1156.7	y_9
1168.7	b_9
1184.7	x_9+2H
1237.6 / 1239.6	a_{10} / a_{10}+2H
1270.7	z_{10}+2H
1282.7	c_{10}
1402.7	a_{11}+2H
1433.8	z_{11}+2H
1445.7	c_{11}
1474.7	x_{11}
1515.8	a_{12}+2H
1543.8	b_{12}+2H
1546.8	z_{12}+2H
1558.8	c_{12}
1575.8	b_{13}-x_{12}+3H
1587.8	x_{12}
1600.8	*non-specific*
1611.8	a_{13}-z_{13}+H
1628.8	a_{13}+2H
1644.8	*non-specific*
1656.8	b_{13}+2H
1672.8	$[M+H]^+$

Table S3: Specific fragments observed in SHI-irradiated bradykinin samples. The nomenclature is the same as used in Tab. S1.

m/z	**Fragment species**
407.2	*non-specific*
530.8	$[M+2H]^{2+}$
545.2	*non-specific*
555.3	b_5
572.3	c_5
585.2	*non-specific*
600.3	*non-specific*
653.3	y_5
710.3	y_6
756.4	c_7
775.4	x_8-c_8+2H
784.4	*non-specific*
807.4	y_7
858.4	a_8
886.4	b_8 / x_8-a_9+H
887.4	z_8
903.4	c_8
904.4	y_8
932.5	x_8+2H
973.5	*non-specific*
999.5	z_9-a_9+H
1016.5	a_9+2H
1042.5	b_9
1043.5	z_9
1060.5	$[M+H]^{+}$

References

[1] Roepstorff, P.; Fohlman, J. Proposal for a Common Nomenclature for Sequence Ions in Mass Spectra of Peptides. *Biomed. Mass Spectrom.* **1984**, *11*, 601.

[2] Biemann, K. Contributions of Mass Spectrometry to Peptide and Protein Structure. *Biomed. Environ. Mass Spectrom.* **1988**, *16*, 99 – 111.

[3] Ziegler, J. F.; Ziegler, M. D.; Biersack, J. P. SRIM - The stopping and range of ions in matter. *Nucl. Inst. Meth. Phys. Res. B* **2010**, *268*, 1818 – 1823.
